# Gammaretroviral Vectors: Biology, Technology and Application

**DOI:** 10.3390/v3060677

**Published:** 2011-06-03

**Authors:** Tobias Maetzig, Melanie Galla, Christopher Baum, Axel Schambach

**Affiliations:** Department of Experimental Hematology, Hannover Medical School, OE6960-HBZ, Carl-Neuberg-Strasse 1, D-30625 Hannover, Germany; E-Mails: maetzig.tobias@mh-hannover.de (T.M.); galla.melanie@mh-hannover.de (M.G.);

**Keywords:** Murine Leukemia Virus, gammaretroviral, split packaging design, SIN vector

## Abstract

Retroviruses are evolutionary optimized gene carriers that have naturally adapted to their hosts to efficiently deliver their nucleic acids into the target cell chromatin, thereby overcoming natural cellular barriers. Here we will review—starting with a deeper look into retroviral biology—how Murine Leukemia Virus (MLV), a simple gammaretrovirus, can be converted into an efficient vehicle of genetic therapeutics. Furthermore, we will describe how more rational vector backbones can be designed and how these so-called self-inactivating vectors can be pseudotyped and produced. Finally, we will provide an overview on existing clinical trials and how biosafety can be improved.

## 1. Introduction

Retroviral vectors are fascinating and efficient delivery tools for the transfer of nucleic acids. As a hallmark, all retroviruses are capable of reverse transcribing their single stranded RNA genome into double stranded DNA, which will be stably integrated into the host cell genome [1]. As highly evolved parasites they act in concert with cellular host factors to deliver their nucleic acid into the nucleus, where they exploit the host cell’s machinery for their own replication and long-term expression occurs. The first approaches of retrovirus-based gene transfer were initiated almost 30 years ago [2–9] giving first evidence that retroviral gene delivery is far more efficient than DNA transfection and that retroviral gene transfer can be used in a murine bone marrow transplantation model. This paved the way for further developments in gene therapy of the hematopoietic system to correct inborn or acquired diseases of the blood and immune system.

During the past 30 years, the biology of the family of the *Retroviridae* has become much better understood. This knowledge allows the rational design of retroviral vectors for specific applications, creating not only more efficient but also safer vector tools. Retroviral vectors have been designed based on various members of the *Retroviridae* including Foamyvirus [10,11], Human Immunodeficiency Virus (HIV-1) [12,13], Simian Immunodeficiency Virus (SIV) [14], Bovine Immunodeficiency Virus [15], Feline Immunodeficiency Virus [16], Equine Infectious Anemia Virus (EIAV) [17], Murine Leukemia Virus (MLV), Bovine Leukemia Virus [18], Rous Sarcoma Virus (RSV) [19,20], Spleen Necrosis Virus (SNV) [21], and Mouse Mammary Tumor Virus [22].

While the other reviews in this Special Issue will cover developments in Foamyvirus-derived and HIV-1 derived lentiviral vectors, this paper will focus on gammaretroviral vectors based on the simple organized MLV. We will summarize the recent developments and build a bridge from retroviral biology and vector design to clinical trials. Furthermore, we will give an overview on safety aspects and discuss which steps need to be taken to further improve efficacy and safety of this vector family.

## 2. The Retroviral Genome and Particle Organization

**Figure 1.**
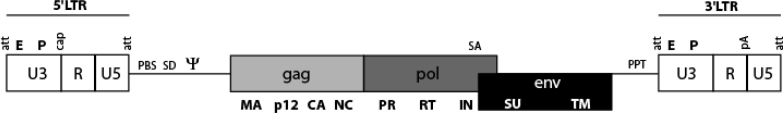
Proviral genome structure of the Murine Leukemia Virus (MLV), a simple retrovirus. Indicated are the 5′ and 3′ long terminal repeat (LTR; open boxes) regions comprising U3, R and U5, as well as open reading frames (filled boxes) for gag, pol and envelope (env) proteins. Processed protein subunits are indicated in bold. att, attachment site; cap, 5′RNA capping site; pA, polyadenylation site; PBS, primer binding site; SD, splice donor; ψ, packaging signal; SA, splice acceptor; PPT, polypurine tract; MA, matrix; CA, capsid; NC, nucleocapsid; PR, protease; RT, reverse transcriptase; IN, integrase; SU, surface; TM, trans-membrane; E, enhancer; P, promoter.

The development of gene therapy vectors originating from pathogenic viruses could only be achieved with deeper insight into their biology, starting with the understanding of genome structure, particle composition and the retroviral life cycle as exemplified for MLV (reviewed in [23,24]). The reverse transcribed and integrated proviral DNA of MLV is flanked by two “incomplete” long terminal repeats (LTR) which are normally structured into U3, R and U5 (Figure 1). Since transcription of the proviral DNA is initiated by the enhancer-promoter located in the 5′ U3 region, the viral genomic RNA starts with R, and is followed by U5, the primer binding site (PBS) for initiation of reverse transcription, the major splice donor (SD) and the packaging and RNA dimerization signal (ψ), all located upstream of the translational start codon of Gag/Pol (encoding structural and replication proteins). Downstream of the gag/pol coding region the env (encoding the viral glycoproteins) reading frame is found, whose expression is enabled by a splice acceptor located in pol. The 3′ untranslated region of the RNA contains the polypurine tract (PPT), and the 3′ “incomplete LTR” consisting of the 3′ U3, and the 3′ R region. The latter contains the polyadenylation signal and is thus followed by a polyA tail. Since genomic viral RNA carries a 5′ cap and a 3′ pA tail, it resembles a cellular mRNA. It is only due to the unique mechanism of reverse transcription that the complete LTRs are restored prior to integration of the virus into the host cell chromatin.

The location of ψ downstream of the major splice donor makes sure that unspliced genomic RNA preferentially associates with viral structural proteins for packaging into budding particles, while spliced subgenomic RNA lacking ψ can only be translated into envelope proteins. Typically more than 50% of the viral transcript is expressed as the unspliced full-length species. Alternative splicing in MLV is regulated at the level of the major splice donor: experimental data support a model according to which a complex RNA secondary structure (stable stem loop structure) is formed upstream of the major splice donor which synergizes with suboptimal duplex formation between U1 snRNA and the major splice donor to attenuate the splicing process [25,26]; in contrast to complex lentiviruses, the splice acceptor upstream of the Env reading frame plays a minor role in the regulation of balanced splicing in MLV.

All *Retroviridae* contain open reading frames encoding for structural (group associated antigen, gag), replication (pol) and envelope (env) proteins which need to assemble in predefined ratios, and require proteolytic processing to form infectious particles. MLV Gag is cleaved during particle maturation by the retroviral protease into matrix, capsid, p12 and nucleocapsid, and similarly Pol into protease, reverse transcriptase and integrase. In contrast, Env as a bipartite transmembrane protein is cleaved by furin within the golgi apparatus while being trafficked to the cell surface [27,28].

Once the virus particle is detached from its host cell, it resembles an enveloped ribonucleoprotein complex that is coated by a packaging cell derived lipid bilayer and envelope proteins. Matrix proteins form a ring-like structure underneath the viral membrane to assist anchorage of envelope proteins as well as targeting of Gag and Gag/Pol precursor into the budding virus. This outer layer disassembles early after cell entry and releases the viral core into the cell cytoplasm. The core is formed by a stable capsid lattice which protects the viral genome from degradation and thus forms the environment for reverse transcription and assists subsequent access of the pre-integration complex (PIC) to the chromatin of the target cell as depicted below.

### 2.1. The Early and Late Phase of the Gammaretroviral Life Cycle

**Figure 2.**
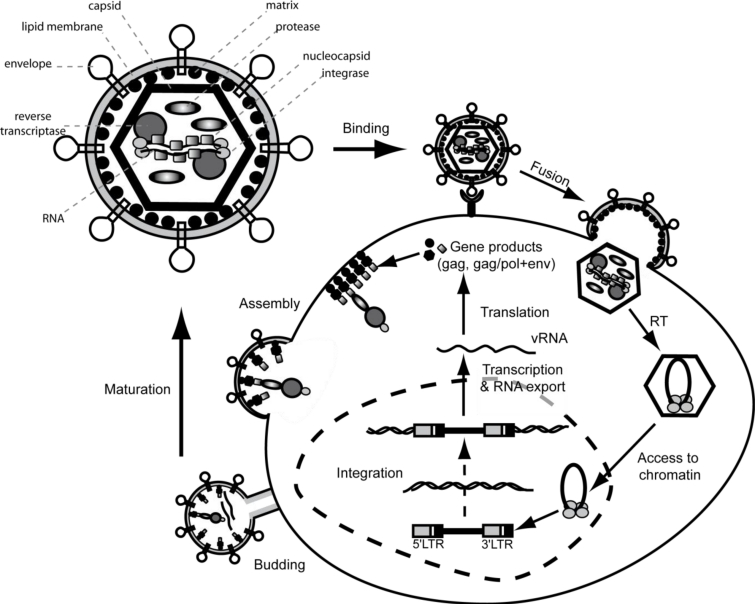
The retroviral life cycle exemplified for MLV. The retroviral particle binds with its envelope to cognate receptors on the target cell surface, which facilitates entry of the virus core into the cell cytoplasm. After reverse transcription, viral DNA is integrated into the host cell chromatin, transcribed, and translated into viral proteins that assemble and bud from the plasma membrane to complete the life cycle with extracellular maturation. RT, reverse transcription; vRNA, viral RNA.

The viral life cycle is divided into an early and a late phase (Figure 2). The early phase begins with the binding of viral envelope molecules to receptors on the target cell surface, which ultimately defines the target cell tropism of the virus. The interaction will trigger conformational changes in the envelope proteins that lead to entry of the virus core into the cell cytoplasm either via fusion of the viral and cellular lipid membranes or by transition through the endosome [29,30]. In both cases the viral RNA genome will be reverse transcribed into its double stranded DNA intermediate within the reverse transcription complex (RTC) comprising the dimeric RNA genome, capsid, p12, nucleocapsid, reverse transcriptase, and integrase in conjunction with cellular proteins [31,32]. It has been suggested that the two copies of the single stranded RNA genome serve as a fail-safe mechanism for production of genomic DNA since defects in one strand can be compensated by using the other strand as a template [33,34]. Alternatively, or in addition, interstrand recombinations can increase the retroviral genome diversity. After formation of the proviral DNA, access to the host cell chromatin is, in case of HIV, mediated by interacting with components of the host cell nuclear import machinery (e.g., TNPO3, RANBP2) [13,14], further facilitated by the presence of complementary nucleophilic elements [35]. In addition, the instability of the lentiviral capsid [36–38] as well as resistance against cellular restriction factors [39] support the transduction of cells independent of their cell cycle status. Since MLV lacks active nuclear import elements and is encapsulated by a highly stable capsid core, it depends on nuclear envelope breakdown and progression through mitosis in the early phase after transduction [40–42].

The integration of the viral genome is mediated by the PIC constituted from integrase, capsid, p12 and proviral DNA, as well as, host cell proteins [43–45]. One such protein, barrier to autointegration (Baf), prevents the intramolecular strand transfer by binding to double-stranded DNA and thus shifts the reaction towards productive integration which is mediated by the retroviral integrase protein in concert with cellular cofactors [46–49]. Since Baf is also found in the PIC of HIV [50], it likely plays a conserved role for retroviral integration. The retroviral integration reaction is a three step mechanism: (I) The invariant CA dinucleotide at the 5′ and 3′ LTRs is removed from the 3′ strand (3′ processing), (II) integrase mediates the strand transfer between 3′ viral DNA and cleaves cellular genomic DNA, and (III) cellular DNA repair proteins remove the two unpaired nucleotides from the LTRs and covalently join free DNA ends between both genomes which finalizes stable integration (reviewed in [51]).

Once proviral DNA is anchored within the host cell chromatin, the “late phase” of viral replication begins with the expression of viral RNA from the 5′ U3 region. Viral RNA can either remain unspliced, which results in full length RNA serving for genome replication as well as template for Gag/Pol translation, or can be subjected to splicing between the major splice donor and acceptor sites located in the leader and the end of pol, respectively. The presence of incompletely processed splice signals on cellular mRNA leads to nuclear retention as a means of quality control which required retroviruses to evolve alternative export strategies harnessing various cellular export routes. The “constitutive transport element” (CTE) of Mason Pfizer Monkey virus binds to the cellular RNA export factor Tap, and HIV’s Rev responsive element (RRE)—Rev interaction hijacks the protein export machinery via binding to Crm-1 [52–54]. Despite a genome size of only about 10 kb, such export elements have not yet been identified in MLV. Experiments with engineered viral genomes suggest that nuclear egress must be independent from gag/pol, env and 3′ U3, and thus must be located elsewhere [25,55,56]. Regardless of the presence of one long open reading frame encoding for gag and pol, viral particles contain ∼10- to 20-fold more gag than pol derived proteins which is crucial for infectivity [57]. In the case of MLV, in 5–10% of transcripts a stop codon at the end of gag is misinterpreted by the translation machinery as glutamine (read-through suppression), and results in a read-through fusion protein [58–60]: Gag/Pol, which is transported and anchored to the plasma membrane via a myristoylation site in the matrix protein [61,62]. Subsequently, membrane localized Gag and Gag/Pol molecules as well as transmembrane envelope proteins assemble at privileged sites of the plasma membrane, so-called “lipid rafts”, which are of transient nature and rich in cholesterol, sphingolipids and phosphoglycerides [63,64]. After assembly, viral particles, still containing uncleaved structural and replication proteins, will bud from the plasma membrane, and during subsequent maturation steps, initiated from the viral protease, will mature into functional monomers of the infectious particle before the replication cycle can start again.

## 3. How to Turn an Infectious Virus into a Vehicle for Delivery of Genetic Therapeutics

**Figure 3.**
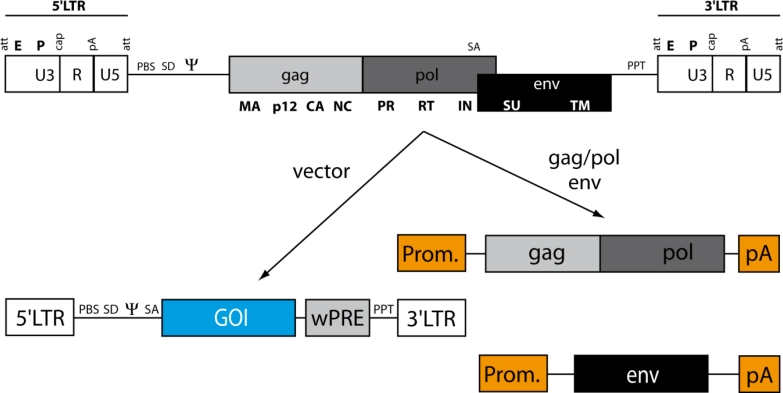
From the virus to the vector. The general genome structure (see Figure 1) is depicted above with LTRs, packaging signal ψ and the open reading frames for structural proteins (gag), replication enzymes (pol) and envelope glycoproteins (env). For construction of a gene transfer tool, the *cis*-acting elements (LTRs and leader region with ψ) and the open reading frames for gag/pol and env are divided onto separate plasmids. Gag/pol and env are placed in a heterologous DNA context (Prom: promoter; pA: polyA signal) that can be delivered as a plasmid transiently or stably inserted into the host cell DNA of the packaging cell. The gag/pol and env plasmids lack ψ, so that the encoded RNA cannot be packaged into retroviral particles. In contrast to the helper plasmids, the vector DNA containing gene of interest (GOI, e.g., a therapeutic transgene cassette), flanked by the LTRs, harbors ψ for efficient packaging into the viral particle.

Due to the potential of retroviruses to utilize receptor mediated transduction of their genetic information into a variety of somatic cells (e.g., embryonic stem cells, hematopoietic and neural stem cells), special interest evolved to harness these mechanisms for therapeutic intervention and the treatment of (mono)genetic diseases. By inserting the gene of interest (GOI, transgene) within the retroviral genome and by employing all retroviral proteins necessary for successful infection, retroviral particles serve as well evolved and specialized “gene ferries”. To take advantage of a virus for vector development and to avoid the generation of replication competent retrovirus (RCR) in gene-modified cells, it is necessary to separate genes encoding for structural and enzymatic proteins (Gag/Pol), as well as the gene encoding envelope proteins (Env) from the retroviral genome. This can be achieved by the so-called split packaging design (Figure 3). The result is a retroviral vector, which still contains the packaging signal (ψ), the primer binding site (PBS) and the long terminal repeats (LTR), but harbors the transgene instead of genes encoding for structural and enzymatic retroviral proteins. The viral Gag/Pol and Env proteins are encoded on separate helper expression plasmids, which lack all other retroviral components including the retroviral packaging signal, and thus lower the probability of recombination events. In contrast to HIV-1 or Foamy virus, the gammaretroviral packaging system does not require the incorporation of any sequences overlapping with coding sequences of gag, pol or accessory genes [10,11,65,66]. Moreover, the gammaretroviral leader sequence of the vector has been liberated from ATG initiating potential ORFs, which minimizes the risk to produce immunogenic peptides derived from the pre-canonical translational initiation [66–68].

Conventional retroviral vectors are driven by the promoter/enhancer sequences of the 5′ LTR (so-called LTR-driven vectors). Within the U3 region the enhancer/promoter sequences are tightly clustered (e.g., various transcription factor binding sites, CAAT and TATA boxes) resulting in relatively strong U3 promoters. However, compared to the standard Moloney MLV sequences, improved variants, e.g., derived from Spleen-Focus Forming Virus (SFFV) and Myeloproliferative Sarcoma Virus (MPSV), could be generated which show higher gene expression in a variety of somatic cells, and especially hematopoietic cells [66,69–71].

In the leader region, the natural splice donor (located at the foot of a large R-U5 loop secondary structure [25,72]) together with the env splice acceptor placed after the packaging signal ψ can be exploited to create a conditional intron (which is transferred into target cells because of the presence of ψ) as originally proposed by Mulligan, Miller and colleagues in the MFG and LN vector designs [73,74]. It could be demonstrated that presence of an intronic sequence in the leader region had a beneficial effect on transgene expression [66], probably because introns can facilitate mRNA export and translatability of the retroviral RNA in target cells [71,75]. As an alternative explanation, the splicing within the retroviral leader removes possibly disturbing RNA secondary structures, which might hamper gene expression and translatability.

The leader does not just only contain ψ, splice signals and secondary structures but also the PBS to which a cellular tRNA molecule binds to initiate reverse transcription (see above). Interestingly, the PBS is also the target of innate immunity and is has been a long mystery as to why the PBS is involved in silencing of retroviral expression in embryonic stem cells and also in some somatic cells. Only recently the underlying cause for this silencing mechanism has been elucidated [76,77], pointing to a broader picture of antiviral immunity in primitive stem cells [78]. Wolf and Goff could show that a protein complex binding to the wild-type PBS of MLV is responsible. This complex consists of TRIM28 (Kap-1), a well-known transcriptional silencer, working in concert with the zinc finger protein ZFP809 bridging proviral DNA and TRIM28. Substitution of this wild-type PBS with a PBS binding the tRNA^Gln^ (instead of tRNA^Pro^), derived from an endogenous retroviral sequence (dl587rev), led to new gammaretroviral vectors which are less prone to silencing in embryonic stem cells and other somatic cell types [69,79–81]. Another interesting principle involving the PBS is the usage of an artificial PBS (aPBS), which does not match any natural occurring tRNA molecule (see also Section 7). Transcomplementation with the matching tRNA primer creates a switch for initiation of reverse transcription and therefore might be used as a safety feature [82].

For the production of gammaretroviral vector particles, both Gag/Pol and Env proteins as well as the retroviral vector construct are either transiently or stably co-expressed in so-called “packaging cell lines” (e.g., human embryonic kidney derived 293T cells). Since *gag*/*pol* and *env* expression constructs lack ψ, viral structural proteins only recognize the ψ-containing retroviral vector construct leading to a preferential packaging of retroviral vector genomes into infectious particles. After entry of the particle into the target cell, only the nucleic acid of the retroviral vector construct is reverse transcribed and stably integrated into the host genome. Since *gag*/*pol* and *env* are only transferred in the form of proteins (and not as nucleic acid) the generation of replication competent retroviral vector progeny is prevented.

Limitations of gammaretroviral vectors may arise from the poor infection of non-dividing cells, faulty reverse transcription, intracellular restriction factors [83,84] and the risk of insertional mutagenesis [85–87] (see also below). Life-cycle related constraints may limit vector design, e.g., the expression cassette should be without introns (with the exception of the leader) or internal polyadenlyation signals and larger secondary structures or repetitive sequences should be avoided because of a possible interference with reverse transcription. Although long expression cassettes (up to 10 kb) can be incorporated into retroviral vectors [69,88,89], it should be noted that the size of an insert is generally a complicating factor for retroviral transgene expression and titer. Moreover, it is important to consider that the chromatin architecture and activity as well as epigenetic modifications in the vicinity of the integration site might influence vector performance [90], and might thus require future optimization of the expression cassette [91].

## 4. Pseudotyping of Retroviral Vectors for Targeting Approaches

As described above, retroviruses enter cells by taking advantage of a defined envelope/host cell receptor interaction which not only mediates specificity but also restricts the virus to a limited spectrum of permissive target cells and species. This behavior can be exploited for targeting approaches, which are summarized in this section (see below and Table 1).

Nature created at least three different strains of MLV that basically share, with respect to gag and pol, the same genome sequence but strongly differ in their envelope proteins. This implies that among the viral proteins, the envelope might have been the one with the least requirement for sequence maintenance or, from another point of view, the one with the highest evolutionary pressure to adapt to new virus—host cell interactions. While ecotropic MLV binds to the murine cationic amino acid transporter (mCAT), amphotropic MLV interacts with a sodium-dependent P_i_ transporter (PiT2), and xenotropic MLV gains access to the cell cytoplasm via the XPR1 receptor (xenotropic and polytropic retrovirus receptor 1) [92–94]. Based on these interactions, the first envelope only mediates transduction of murine and rat cells while the latter two facilitate transduction of a broader species spectrum including humans.

This example shows two interesting observations. (1) Target cell tropism is mediated by the virus envelope and (2) the same virus can be equipped with different envelopes and thus acquires different tropisms. In the context of vector development and gene therapy, the exchange of the natural envelope protein of any given virus/vector—so-called pseudotyping—is of great importance for the efficacy of gene transfer since it allows to choose the optimal envelope for a certain application. As a rule of thumb, the pseudotype should be chosen according to the expression level of its receptor on the target cell: the higher the receptor level, the higher the gene transfer rate. Consequently, human HSCs can easily be transduced with RD114 (a simian endogenous retrovirus) pseudotyped viruses [95], while amphotropic viruses or viruses equipped with the envelope of Gibbon Ape Leukemia Virus (GALV), both binding to phosphate transporters (PiT2 and PiT1, respectively) show higher transduction rates of more mature hematopoietic cells [96–98]. Despite their suboptimal performance on human HSC, ampho and GaLV pseudotyped MLV were successfully used in an X-SCID gene therapy trial conducted in Paris and London (see below).

The sixth important pseudotype is the glycoprotein (G) of Vesicular stomatitis virus (VSVg). This envelope has got several advantages over the others in that it is very stable and thus allows for concentration of viral particles by high speed ultracentrifugation [99]. In contrast to RD114, GaLV and other pseudotypes, VSVg can also be efficiently used for pseudotyping of retroviral vectors other than MLV without the requirement for additional modifications of its cytoplasmic tail [100–102]. Although VSVg transduces a variety of cells from different species, its application in humans is limited by its sensitivity to complement inactivation when systemically applied [100]. In the future, it will be of importance to develop improved *in vivo* gene transfer strategies, not only to expand therapeutic cancer intervention but also to facilitate gene therapy of organs that cannot be modified by *ex vivo* gene therapy (e.g., the heart). The feasibility of *in vivo* cancer gene therapy was recently tested in phase I/II clinical studies with a pathotropic (an envelope that recognizes diseased cells) retrovirus for targeted killing of transduced cells [103,104]. Although systemic application of this vector seemed to be safe, it was restricted to natural targets of von Willebrand factor whose matrix binding elements had been incorporated into the ecotropic MLV envelope. An alternative targeting strategy is derived from modified measles virus envelopes. Their unique feature is the separation of the receptor binding (H; *hemagglutinin)* and fusion (F) function to two envelope subunits. Although H of wild-type measles virus binds to SLAM/CD150, a surface molecule expressed on lymphocytes, “blinding” of H for its receptor in combination with incorporation of a specific targeting domain (e.g., a single chain antibody) mediates cell type specific transduction [105–107]. This offers a greater freedom over previously described targeting strategies [103,108,109]. It will be interesting to see to what extent this kind of newly evolving targeting technology will boost the *in vivo* application of gene therapy.

In addition to the aforementioned pseudotypes, it is of note that MLV could also be equipped with envelopes of Hepatitis B virus [115], Lymphocytic Choriomeningitis Virus (LCMV) [116] and others, which shows, although this list is by far not complete, the wide spectrum of pseudotypes suitable for MLV production.

## 5. SIN Design and other Vector Modules

**Figure 4.**
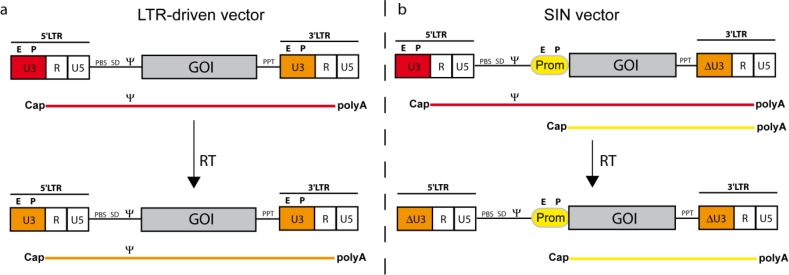
Schema of a gammaretroviral LTR-driven vector (**a**) and a SIN (self-inactivating) vector (**b**). The upper part shows the plasmid configurations along with the corresponding transcripts originating from either the internal promoter (Prom; b) and/or the LTR (a and b). The lower part depicts the vector architecture after reverse transcription (RT; *i.e.*, in the integrated form). Note that the promoter from the 3′LTR in the plasmid configuration is copied into the 5′LTR during reverse transcription facilitating the duplication of either SIN deleted U3 (ΔU3) or regular U3 promoter sequences. The corresponding RNAs (with Cap and polyA) are depicted under the vector diagrams (color codings indicating the appropriate promoter).

Although the generation of LTR driven vectors was a milestone in terms of vector development, this architecture was still not optimal, because it harbored two promoters and no option to modulate gene expression. As a consequence, Eli Gilboa and colleagues developed the first retroviral SIN (self-inactivating) vector in 1986 [117]. Low vector titers could only be overcome by the replacement of the 5′ U3 region with strong promoters (needed in the packaging cell line) derived from Cytomegalovirus or RSV. Titers could further be increased by adding an SV40 enhancer upstream of the RSV promoter and by inclusion of a post-transcriptional regulatory element derived from the woodchuck hepatitis virus upstream of the 3′ SIN LTR [13,56,118]. SIN vectors harbor a deletion within the 3′ U3 region initially comprising enhancer/promoter activity. During reverse transcription, this deletion is copied to the 5′ LTR, depriving the provirus from LTR-located promoter activity and thus conferring transcriptional control to an internal promoter of choice (see Figure 4). The SIN design has multiple advantages. First, it reduces the risk of RCR formation, impedes the mobilization of vector sequences in case of wild-type virus infection or RCR superinfection and increases the autonomy of the internal promoter. Second, the deletion of the promoter /enhancer elements and the incorporation of a cellular (more physiological) internal promoter create safer vector tools and reduce the risk of insertional upregulation of neighboring genes (see Section 6 below). Third, the choice of the internal promoters can be made according to specific requirements of gene expression needed for a given application (e.g., tissue-/lineage-specific or regulated). In order to maximize gene expression and therapeutic outcome, early generation SIN vectors often made use of strong ubiquitously active viral promoters. Later generations focused on utilizing weaker (cellular) promoters in combination with codon-optimized transgene cassettes (see below) to compromise between genotoxicity and expression intensity. As an alternative, a subset of retroviral promoters, e.g., derived from human endogenous retroviruses (HERV) can also be utilized that have evolved as transcriptional control elements for cellular gene expression and differ widely in activity and tissue specificity [119].

In addition to transcriptional control by cell type or lineage specific promoters, transgene expression can be regulated on the posttranscriptional level. An important concept is the incorporation of repeats of microRNA target sites into the transgene harboring retroviral RNA [120,121]. In this way, cells which express the corresponding microRNA downregulate transgene expression. Potential applications include “posttranscriptional detargeting” from antigen-presenting cells or hematopoietic stem cells [122,123]. On the other hand, retroviral transcripts can also be stabilized. The incorporation of the woodchuck posttranscriptional regulatory element (wPRE) increases mRNA stability, mRNA export and translatability [71,118,124]. Also the codon optimization of the transgene sequence goes along these lines and increases mRNA stability and translatability of the retroviral RNA [125,126]. A third possibility to increase gene expression from retroviral transcripts is to improve their polyadenylation. Due to the compact structure and the redundancy of the LTRs, orthoretroviruses have relatively weak polyadenylation signals [127]. Their polyadenylation efficiency further decreases in SIN vectors, where parts of the U3 regions, which have been shown to stimulate polyadenylation, are deleted [128]. Interestingly, upstream polyadenylation enhancer sequences (USE elements) have also been identified in other viruses (e.g., SV40), and can thus be incorporated into retroviral vectors to enhance their polyadenylation efficiency [129], possibly by recruiting more polyadenylation factors to the correct polyA site.

Taken together, the generation of SIN vectors and improvement of their post-transcriptional gene expression allow usage of weaker internal promoters. Thereby, this vector family creates a more efficient and possibly also safer tool for gene therapy (see also Section 6 and outlook). However, all integrating vectors harbor a residual risk for insertional upregulation of neighboring alleles or disruption of cellular genes. Consequently, non-integrating vectors (which are reviewed in Section 7) represent interesting tools for transient retroviral gene transfer avoiding the integration step and thus the major risk of insertional transformation.

## 6. Towards Clinical Production of Retroviral SIN Vectors

In general, retroviral vectors can be produced in two ways: transient or stable. In the former the vector and helper constructs are delivered transiently as plasmids, while in the latter all components are stably integrated into the host cell genome of the packaging cell line. The biggest advantage of transient production is that it is more flexible and that it avoids the time consuming process to generate stable vector producing packaging cells. Special consideration of the design of vectors and corresponding packaging allowed clinical grade vector production with titers of ∼10^6^–10^7^ infectious particles/mL [130]. Of note, transient production in 293T cells is also available for other retroviral vectors, such as lentiviral SIN vectors.

In contrast, stable packaging cells are more difficult to be generated, which is still a very hard task as for lentiviral vectors, since not all components (e.g., VSV glycoprotein and lentiviral Gag/Pol) are well tolerated within the packaging cell line over extended periods of time. Stable packaging cell lines for conventional LTR-driven gammaretroviral vectors have been produced. They could be established by transducing existing stable packaging cell lines (e.g., PG13 [131], harboring gammaretroviral gag/pol and GALV env) with LTR-driven gammaretroviral vectors and selection for the best producer clones. However, this procedure is not adaptable to gammaretroviral SIN vectors, which are—due to the self-inactivating U3—not capable of producing packageable genomic viral RNA in transduced packaging cell lines.

To circumvent this problem, the recombinase-mediated cassette exchange technology (RMCE; [132,133] was employed to tag and exchange a highly expressing, open chromatin locus with the desired vector construct [88,134]. Here a site-specific recombinase (Flp) was used in concert with two heterospecific (non-interacting) Flp recognition sites (FRT) to target the packageable retroviral SIN vector construct into the predefined locus. This strategy can produce retroviral SIN vectors with titers in the range of 10^5^–10^6^ infectious particles/mL and proof-of-concept has been demonstrated as for the clinically relevant vectors expressing collagen 7 (epidermolysis bullosa; [88]) as well as IL2γc (severe combined immunodeficiency) and gp91phox (chronic granulomatous disease) [134].

## 7. Exploiting Intermediate Steps for Developing Tools for Transient Genetic Manipulations

**Figure 5.**
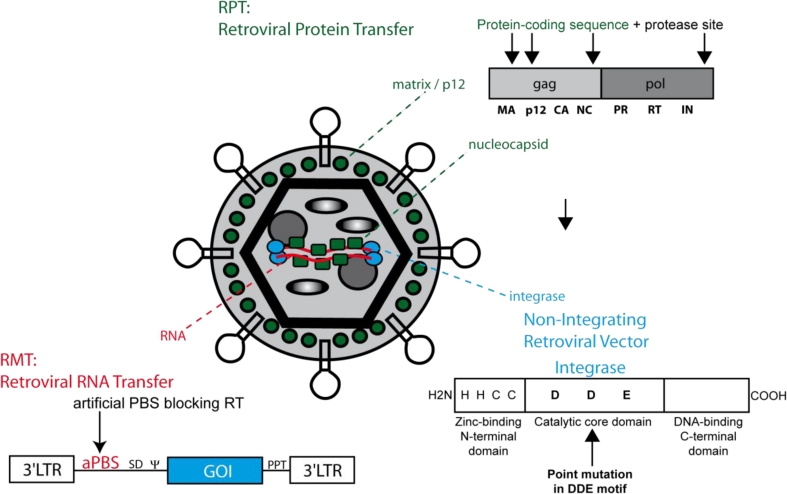
Retroviral tools for transient genetic manipulation. A color-encoded retroviral particle is shown. The following 3 retroviral techniques taking advantage of retroviral features are demonstrated. As for RPT (retroviral protein transfer, indicated in green) the coding sequence for a protein of interest is embedded into tolerant sites of the gag or pol ORF (e.g., MA, p12, NC or IN). To liberate the protein of interest during retroviral maturation the protein of interest is flanked by an additional protease site. As for RMT (retroviral mRNA transfer, red) the retroviral mRNA is modified by an artificial primer binding site not matching any natural occurring tRNA but can still serve as a template for immediate translation. The majority of non-integrating retroviral vectors (see blue) have a point mutation within the catalytic core domain of integrase (see structure). Although integration is blocked the retroviral side products 1- and 2-LTR circles (episomal forms) can still serve for transient gene expression.

For some applications, the transient/short-term expression of a given protein is sufficient or even—due to cytotoxic side effects—required. But how can an integrating and permanent expressing retroviral gene vehicle be turned into a transient expression tool? The knowledge of the retroviral replication cycle allows defined interventions avoiding the (potentially harmful) integrating nature of retroviruses. Considering retroviral particle composition and steps within the early phase of the retroviral life cycle three different possibilities are thinkable: Episomal DNA, delivery of RNA or protein (Figure 5). The following section will provide an overview of these different options.

To date the most common way to achieve sufficient gene expression from retroviral vectors while avoiding their stable integration into the host’s cell genome is the use of gammaretroviral and lentiviral integration-deficient vector particles. In theory, integration can be abrogated by blocking the integrase attachment site on the viral LTRs, using an integrase inhibitor (e.g., raltegravir) or—as mostly used—by modifying the integrase itself. The retroviral integrase consists of an N-terminal zinc finger domain followed by a catalytic core domain and a C-terminal DNA-binding domain. In contrast to wild-type particles, the latter are packaged with Gag/Pol variants whose integrase has been inactivated by the introduction of specific point mutations within the DDE motif of its catalytic domain. The DDE motif of the HIV integrase is located at positions D64, D116 and E152 [135], the DDE motif of MLV integrase at D125, D184 and E220. Point mutations that result in amino acid changes at these positions specifically inhibit integration but not other intermediate steps of the retroviral life cycle (e.g., cellular and nuclear entry, reverse transcription) and result in extrachromosomal (episomal) viral DNA, which may serve as a transient source of transgene expression. DNA repair processes form 2-LTR circles, and homologous recombination may lead to 1-LTR circles. The most common mutation for lentiviral vector systems is D64V, for which efficient gene expression especially in non-dividing cells (such a retina cells) could be demonstrated [136,137]. Whereas episomes are diluted in rapidly dividing cell populations, expression can last long-term in non-dividing cells if the encoded protein is neither toxic nor immunogenic. In analogy, corresponding mutations in the DDE motif of the core domain of MLV have been identified (e.g., D184A) [138] and used in non-integrating gammaretroviral vector systems [139]. Nevertheless, these particles still require cell division to mediate transgene expression. However, the destruction of the catalytic core domain of the retroviral integrase does not prevent spontaneous integration. Detailed analyses revealed that these events are based on non-homologous or homology-driven integrations rather than mediated by potential residual activity of the mutated viral integrase. Although the frequency of residual integration events typically does not exceed 3%, it should be considered that some transient gene therapeutic approaches require provirus-free genomes (e.g., the reprogramming procedure and other applications including oncogenes). So how can residual integrations be further reduced or even circumvented? Our laboratory developed gammaretroviral particles that were modified to transduce either RNAs (retrovirus particle-mediated mRNA transfer, RMT) or proteins (retrovirus particle-mediated protein transduction, RPT) into a given target cell [140,141]. The RMT technology takes advantage of the two capped and polyadenylated retroviral RNA genomes that are embedded in the retroviral particle. To allocate these transcripts resembling cellular mRNAs for translation, reverse transcription needs to be blocked either by chemical intervention (e.g., AZT, a reverse transcriptase inhibitor) or by the inactivation of either reverse transcriptase or the PBS, *i.e.*, the landing platform for the tRNA primer initiating RT. For RMT, the incorporation of an artificial PBS (aPBS) not matching any naturally occurring tRNA molecule, originally designed as a safety feature of the packaging technology for retroviral vectors [82] has been most effective in mediating transient expression of a gene of interest from the retroviral transcript. Compared to expression levels from non-integrating episomal vectors, RMT mediates more rapid but lower expression, and lacks any residual integration events. Of note, in comparison to first generation RMT constructs, improved vector design mediated a further increase in titer and expression allowing to establish a transient and efficient recombinase transfer technology [142].

Another possibility for transient cell modification is the usage of retroviral particles for (ectopic) transfer of proteins. Footprinting approaches could identify tolerant sites in the retroviral genome where coding sequences of heterologous genes can be incorporated [143–146]. Taking into consideration that every particle harbors between 3000 to 5000 gag molecules one can imagine the expected heterologous protein load. Tolerant sites were mapped in MA, p12 and NC, and also modifications of pol (e.g., IN), the latter with the limitation of only a few dozen molecules being incorporated per particle, are well tolerated. This strategy also allows for fluorescent tagging of retroviral proteins. Of note, the fusion to a specific retroviral protein couples the fate of the heterologous protein to its fusion partner (including subcellular localization and degradation). To circumvent this, we introduced an additional protease cleavage site between the protein of interest and the retroviral protein. The retroviral protease thus cleaves the protein of interest from the retroviral protein during extracellular maturation of the retroviral particles, and after transduction of target cells the protein of interest is liberated to follow its designated function. Interestingly, retroviral particles can also be labeled extracellularly (a procedure the authors called “painting”) using fluorescent proteins attached to a modified GPI anchor [147], and serve for cytokine display [148–150].

A comparison of RMT, RPT and non-integrating retroviral vectors shows that all techniques have advantages and disadvantages. While non-integrating episomal vectors give the most robust and longest expression, their application is limited by the risk of residual integrations. RMT is especially suited for applications where low and transient gene expression mediates a strong phenotype (e.g., the application of recombinases). RPT, in contrast to the previous techniques, transfers recombinant proteins instead of viral nucleic acids, and therefore mediates short-term modification of cells without the risk of inducing antiviral immunity against viral genomes. The big advantage of all three retroviral options is that they can be targeted via specific receptors. Here it is important to note that the recently developed measles virus pseudotypes [113,151] allow cell specific targeting for lentiviral and gammaretroviral vectors through the introduction of specific binding sequence (e.g., single chain antibodies) to the H protein of the measles envelope.

## 8. Clinical Applications and How to Improve Safety

### 8.1. Human Gene Therapy: First Steps and Stumbles

Although retroviral gene therapy only represents ∼20% of all gene therapy trials (for a complete overview of human gene therapy trials see [152]), it has a broad application to fight all kinds of diseases (see also selection in Table 2).

One of the preferred targets for retroviral gene therapy is the hematopoietic system. It is maintained by a limited number of hematopoietic stem cells (HSC) that reside within the bone marrow, and harbor the potential to self-renew and differentiate into all blood lineages [153]. Under steady state hematopoiesis, HSC cycle infrequently with extended periods of dormancy between cell divisions, which helps protecting their regenerative capacity [154–157]. Under regenerative stress, e.g., chemotherapy, irradiation or after bone marrow transplantation, repopulating HSCs re-enter mitosis until their niche is replenished, and normal hematopoiesis is restored [154]. This behavior is routinely harnessed in allogenic bone marrow transplantation for the treatment of immunodeficiencies or cancer, where CD34^+^ cells are purified from the bone marrow or peripheral blood of the donor prior to transplantation [158].

In fatal blood disorders where no suitable donor is available, infusion of gene corrected autologous HSC might be the only treatment option, and requires an additional phase of *ex vivo* cultivation which facilitates corrective gene transfer [159–162]. Only recently, cytokine cocktails and signaling pathways have been identified that assist *in vitro* stem cell proliferation while preserving stemness, and might thus increase the success of HSC gene therapy due to enhanced repopulation capacity of the graft [163–166].

In general, gene therapy targeting HSCs involves four major steps: (1) purification and cultivation of HSCs, typically in a mixture with more mature progenitor cells, (2) retroviral gene transfer *ex vivo*, typically followed by at least a few hours of cultivation prior to cell harvest, (3) conditioning of the patient (unless the gene-modified cells have strong selective advantage), and (4) transplantation of gene modified cells. The conditioning protocol is required to create space in the bone marrow of the patient so that the newly transplanted cells will engraft into the niches for subsequent multiplication. In certain protocols, this step is not included due to the enhanced fitness or a survival advantage of the genetically corrected over the diseased stem cells, which will facilitate their engraftment *per se*.

Gene therapy for the treatment of monogenetic diseases is only feasible when a justified risk-benefit assessment is applied, *i.e.*, when the disease is fatal, no other treatment option is available and efficacy and safety have been sufficiently tested in pre-clinical studies [167]. In the context of hematopoietic gene therapy, these requirements are fulfilled by a number of diseases that affect the immune system, the so-called “severe combined immune deficiencies” (SCID). Common to all SCID patients is an impaired immune system, frequent and often chronic infections, and a low life expectancy in the absence of suitable bone marrow donors [168]. In SCID-ADA, the second most common form of SCID, a single enzyme—adenosine deaminase (ADA)—is malfunctional due to a single autosomal gene defect on chromosome 20 that is recessively inherited. As a result of ADA deficiency, cells are impaired in purine metabolism and subsequently toxic levels of dATP accumulate in lymphoid progenitors and interfere with their maturation, and are responsible for neurologic impairment and skeletal abnormalities. Without bone marrow transplantation or bovine PEG-ADA drug replacement therapy, patients have a life expectancy of ∼one year. Although PEG-ADA treatment mediates improved purine metabolism and thus increases the life span of the patient, long-term immune restoration is often not achieved due to the formation of neutralizing antibodies against the enzyme.

In 1990, Anderson, Blaese, and Culver were the first to test gene therapy in the context of SCID-ADA [169]. The disease qualified for this treatment since the addition of a single copy of a functional gene into patient derived lymphocytes should alleviate the phenotype. In 1992, Claudio Bordignon and co-workers also used retroviral vectors to transfer the ADA gene but this time targeted the HSC [170]. In contrast to the first study, where a short lived mature cell population had been genetically modified, gene transfer into HSC was supposed to grant long lasting ADA expression in all progeny cells. Although the clinical benefit of both studies was rather limited, especially due to low gene transfer rates, they proved that retroviral gene transfer into different blood cell types was feasible, and gene modified autologous cells would persist in the patient’s blood circulation without causing adverse effects. These initial observations paved the way for very successful studies by Aiuti and colleagues, where nonmyeloablative bone marrow conditioning facilitated efficient engraftment of gene modified HSC [171]. Until now, their study has enrolled more than 15 patients that were successfully treated and mainly became independent from PEG-ADA drug therapy. Even 10 years after the first patient was treated, no severe adverse effects have been observed so far, which renders SCID-ADA an example for the successful development of gene-therapy medicinal products [172].

Another successful clinical trial including 20 boys had been initiated for the treatment of SCID-X1 in Paris and London some 10 years ago [159,160]. In this disease, a gene defect of the X-chromosome encoded interleukin 2 common gamma chain (IL2γc), which is also part of the cytokine receptor complex for IL4, IL7, IL9, IL15 and IL21, results in the production of dysfunctional B-cells and the complete absence of T- and NK-cells. In 17 of the 20 infant patients, their condition was improved by transplantation of autologous gene modified HSC, while older patients, most likely caused by impaired thymopoiesis, did not respond as well to the therapy [159,160,173]. This might clearly argue for therapeutic intervention in juvenile patients.

After these initial encouraging results, five patients presented with T cell acute lymphoblastic leukemia (T-ALL) two to six years after therapy, with vector integrations in the vicinity of well characterized proto-oncogenes [86,174]. Similar adverse events were observed in two additional gene therapy trials for X-chromosome linked chronic granulomatous disease (X-CGD) and Wiskott-Aldrich syndrome (WAS).

CGD patients suffer from chronic infections due to the genetic deficiencies in proteins belonging to the NADPH oxidase complex, which is responsible to produce reactive oxygen species (ROS) in phagocytic cells. As a result, engulfed pathogens are not destroyed by ROS but persist as intracellular parasites protected from immune recognition. Therefore, CGD patients require permanent antibiotic and antimycotic prophylaxis to minimize periods of life threatening infections. In order to restore immune competence in patients lacking suitable bone marrow donors, autologous HSC have been gene corrected to restore expression of gp91phox, a protein defective in a X-linked form of CGD [161]. Patients experienced a (transient) therapeutic effect. However, in a clinical trial using an LTR-driven gammaretroviral vector, progressive silencing of the transgene cassette compromised the therapeutic effects, and long-term marking was complicated by insertional upregulation of the MDS/EVI1 proto-oncogene, ultimately inducing a myelodysplastic syndrome [87,161,175]

In Wiskott-Aldrich syndrome, the phenotype is induced by a lack of functional WAS protein (WASP) expression in lymphocytes resulting in immunodeficiency and thrombocytopenia. Patients not qualifying for bone marrow transplantation often succumb to infections or bleedings early in life. In a clinical study in Hannover, Germany, several patients were transplanted with gene modified autologous HSCs [162], and despite a clear clinical benefit and polyclonal hematopoiesis, vector induced oncogene upregulation led to T-ALL in one patient [176]. As in the other clinical trials in which severe adverse events were observed in association with insertional proto-oncogene upregulation, an LTR-driven gammaretroviral vector was used in this first clinical trial to treat WAS.

Regardless of a multitude of gene therapy trials aiming to treat hematopoietic disorders, HSC gene therapy can also be used to ameliorate neurodegenerative diseases. This has been effectively shown for the treatment of X-linked adrenoleukodystrophy (ALD), a disease characterized by destruction of brain glial cells and subsequent demyelinization and mental retardation. Patients develop symptoms early in life and most often do not survive their teens. ALD can be effectively treated by bone marrow transplantation when early diagnosed, so that monocyte derived cells can replace neuronal microglia in the brain before the disease gets untreatable. In a recent clinical study, ALD gene transfer mediated by a lentiviral SIN vector into autologous HSC effectively restored glial cells in the brain without disturbing regular hematopoiesis or the induction of adverse events [177]. However, this lentiviral SIN vector used an internal promoter derived from an MLV U3 region, and thus long-term observation may be important to monitor the potential emergence of clonal imbalance. The scope of retrovirus mediated gene therapy could also be expanded to the treatment of fatal skin disorders. In a recent proof-of-principle case study, restoration of Laminin 5 expression in keratinocyte stem cells facilitated *ex vivo* expansion of skin patches, and their engraftment on a Epidermolysis bullosa patient who suffered from blistering and infections of the skin [89,178]. Transplanted areas remained healthy and significantly helped to improve the condition of the patient. Remarkably, in contrast to HSC gene therapy, transplantation of gene modified skin stem cells might be safer since clonal skin grafts can be analyzed prior to transplantation for suspicious insertion events and can routinely be analyzed for signs of cancer after engraftment by visual inspection.

Based on adverse events observed in dose controlled clinical trials for SCID-X1, X-CGD and WASP, it seems ironic that gene therapy trials with replicating retroviral vectors have been launched. These vectors are normally taken to carry killer genes into malignant target cells and help fighting the disease. Although encouraging results could be obtained with these vectors [103,104,179], they are beyond the scope of this review and will therefore not be discussed to a greater extent. However, they clearly show that the use of retroviral vectors as therapeutic agents is constantly expanding and might even grow with the increasing understanding of their biology.

### 8.2. Insertional Mutagenesis

As mentioned above, clinical trials for X-SCID, X-CGD and WAS have unfortunately shown that gene therapy can induce severe adverse effects caused by insertional mutagenesis with subsequent accumulation of secondary mutations and unrestricted proliferation of cells [86,87,174]. In the early days of gene therapy, addition of new genetic information into the genome of the target cell was considered to be safe, since malignant transformation of human cells should require multiple sequential fitness enhancing genome alterations [187] (and references therein). The number of vector integrations per target cell was kept low to avoid adverse effects [188,189]. However, subsequent studies revealed that insertional mutagenesis by a single vector integration in the vicinity of a crucial proto-oncogene may trigger transformation of cells by inducing a clonal selective advantage with accelerated cycling rates, ultimately resulting in clonal expansion not obeying to environmental growth limiting cues. A far more frequent outcome of vector insertion is a mild growth advantage of cells that successfully repopulate the host but still obey to homeostatic mechanisms (benign clonal dominance) [190]. Of note, benign clonal dominance may also reflect a steady state condition normally acquired after transplantation of a limited number of gene-modified HSCs [191].

**Figure 6.**
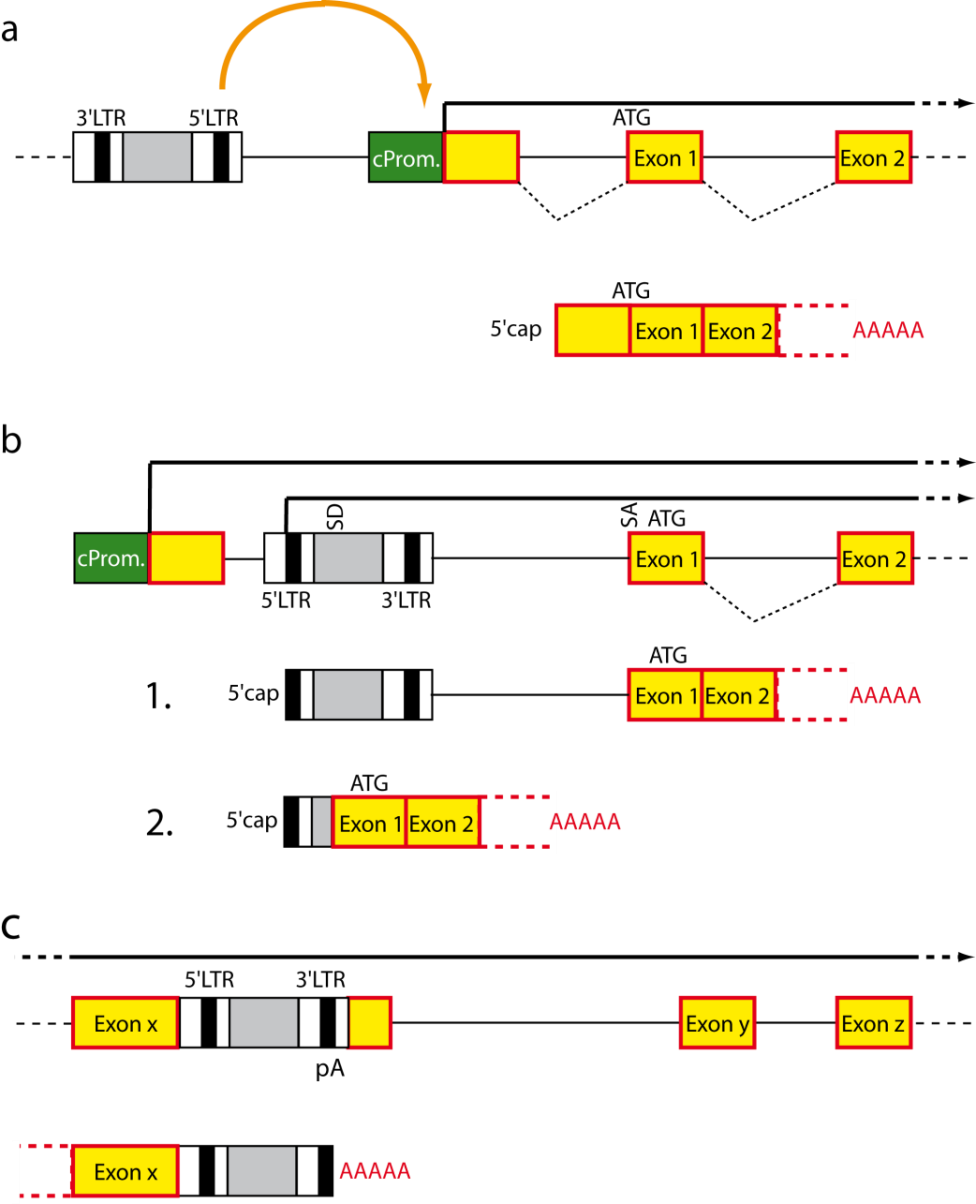
Modes of insertional mutagenesis. (**a**) Vector insertion in reverse orientation upstream of a cellular gene leads to enhancer-mediated upregulation of cellular gene expression. (**b**) Integration between the promoter and the first coding exon uncouples the cellular transcription unit from its promoter and facilitates expression from the viral promoter. Read-through the 3′ pA signal of the virus leads to fusion transcripts that can either remain unchanged or undergo splicing. (**c**) Integration into a transcription unit can mediate pre-mature termination due to usage of pA signals located within the proviral DNA. Green box, cellular promoter; red box, coding exon; LTR, long terminal repeat; ATG, start codon; SD, splice donor; SA, splice acceptor; A_n_, pA stretch.

In a best case scenario, vector insertions do not influence cell behavior which requires insertions ideally to occur in intergenic regions. However, due to the semi-random insertion profile of retroviral vectors, selective intergenic insertions may be difficult to obtain: Gammaretroviruses cluster in the proximity of the transcriptional start site, CpG islands and DNaseI hypersensitive sites, lentiviruses integrate within the whole transcription unit, and alpharetroviruses locate relatively uniformly within the whole genome [192–197]. Consequently, deregulation of gene function and activity will greatly depend upon the nature of the control elements present in the integrated transgene sequence. For an overview of the modes of insertional mutagenesis see Figure 6 and [198].

### 8.3. Enhancer Interaction

Cellular gene expression requires the interplay between promoter and enhancer elements which can be located more than 100 kb away from each other (reviewed in [199]). Cell type specific gene expression is achieved by the binding of specific sets of transcription factors to the enhancer and subsequent *cis* activation of the neighboring promoter(s), which might involve the formation of a DNA loop between both elements, facilitating long range interactions.

Gammaretroviral vectors are prone to mediate insertional mutagenesis by enhancer interactions when they integrate in the vicinity of cellular promoters and provide strong enhancer elements that are bound by ubiquitous transcription factors, and thus override cellular transcriptional control mechanisms. This mode of action requires the vector to integrate in antisense or sense orientation 5′ or 3′ to a cellular gene so that the cellular promoter is activated by the viral enhancer located in the 5′ LTR which is only active when not transcribed (unlike the 3′ enhancer). As a consequence, cellular gene expression will be augmented, and as in the case of integrations close to the promoters of proto-oncogenes like MDS1/Evi1 can lead to malignant transformation [87].

### 8.4. Promoter Insertion

Provided vector integration occurs between the promoter and the first coding exon, transcription of the cellular gene will be uncoupled from its promoter, and will instead be governed by the viral 5′ LTR. Subsequently, a fusion transcript between the viral and cellular transcription unit is generated due to read-through the 3′ pA signal of the viral LTR. The fusion transcript can either contain the whole proviral sequence or can be subjected to splicing events between the viral major splice donor located upstream of the packaging signal and a splice acceptor site upstream of the host gene start codon. In both cases, gene products will contain the whole cellular coding sequence, and will thus give rise to an increased amount of full-length proteins [200,201].

### 8.5. Pre-Mature Termination

In contrast to vector insertions close to transcriptional start sites, insertions within the transcription unit might result in truncated transcripts. This is mainly due to the presence of pA signals in the R regions (when in sense orientation to the transcribed gene), and also cryptic pA signals in the viral enhancer have been identified (in antisense). Truncated transcripts frequently lack regulatory sequences important for mRNA stability, or might encode for hyperactive or dominant negative protein mutants. In all cases cellular homeostasis can be impaired, which for example led to deregulation of HMG2A and subsequent clonal dominance in a recent gene therapy trial for beta-thalassemia [182].

Despite the induction of vector mediated leukemic transformation events in at least three different gene therapy trials (SCID-X1, X-CGD and WAS) with LTR-driven gammaretroviral vectors, the number of loci that mediated insertional mutagenesis was rather limited, and therefore resembles the integration preferences of the vector as well as a limited number of loci capable to drive transformation. In the SCID-X1 trial, four leukemia clones harbored vector insertions in the LMO2 locus and subsequent increase of its expression, while in a fifth patient the upstream region of the CCND2 oncogene was hit [86,174]. A similar LMO2 insertion was found in the leukemia patient from the WAS trial. The insertions found in the 2 patients from the X-CGD trial were located in MDS1/EVI1, PRDM16 or SETBP1 and resulted in a pre-myelodysplastic syndrome in combination with the acquisition of monosomy 7 [87,161].

In accordance with initial considerations most patients not only harbored vector insertions with mutagenic potential but also showed secondary genetic alterations most likely important to facilitate malignant transformation. Except for one patient, all patients with T-ALL could be successfully treated with conventional chemotherapy.

It remains elusive why gene therapy for the treatment of SCID-ADA seems to be rather safe, while in other diseases insertional mutagenesis led to malignant transformation. One reason might be the disease phenotype itself. In contrast to SCID-ADA, primitive hematopoietic or lymphoid cells in X-CGD and SCID-X1 may be subjected to increased replication stress and thus be more prone to the acquisition of secondary growth promoting mutations.

Consequently, malignant transformation in gene therapy might depend on insertional mutagenesis as well as genetic predisposition and disease background, which underlines the need for better disease models and vectors with reduced genotoxicity.

## 9. Conclusion and Outlook

Retroviral gene therapy has proven its effectiveness in a number of recent clinical trials. However, the occurrence of leukemia and premyelodyplasia (in otherwise successful) clinical trials reminded us that safety of gene therapy with integrating vectors needs to be further improved. In this regard, the observed severe adverse events were probably attributable to insertional upregulation of neighboring proto-oncogenes by the strong enhancer/promoter sequences of the LTRs. Here SIN vectors with carefully chosen internal promoters represent safer alternatives making insertional mutagenesis a less likely event [202–204]. Also the use of new cytokine cocktails might be promising to further reduce the likelihood of clonal outgrowth [205].

An important avenue and challenge for gene therapy with integrating (retroviral) vectors will be the development of better assays to predict more precisely insertional adverse events (including upregulation of neighboring genes and also gene disruption), ideally both in cell culture based systems [202,206] and small animal models [190,204], to allow a good risk-benefit assessment of the planned intervention.

As a first consequence of the reevaluated risk-benefit analysis in the X-SCID trials, new clinical trials will be initiated for SCID-X1 in 2011 as part of a multicenter clinical study in Boston, Los Angeles, London and Paris. In this study a gammaretroviral SIN vector with weak enhancer/promoter elements (having a reduced likelihood to mediate insertional transformation by enhancer interactions) will be used, and vector expression is enhanced by the inclusion of the wPRE in the 3′ untranslated region [207]. Another important option to improve vector performance without triggering interactions with neighboring alleles is the codon-optimization of the therapeutic cDNA, thus improving post-transcriptional mRNA processing and allowing the incorporation of relatively weak but tissue-specific promoters [126]. Finally, insulator elements could be placed into the U3 region, to prevent long-distance interactions originating from residual enhancer located in the vector sequence [208].

Although this is a review about gammaretroviral vectors, which—as described here—have clearly improved over the recent years and are far away from the conventionally used MLV derived vector, it should be noted that vector improvements within this and other family members of the *Retroviridae* have cross-fertilized the general usage of retroviral vectors. The infection of non-dividing cells with lentiviral vectors (see review by Cimarelli [209]) is an advantageous feature for infection of slowly or nondividing tissues of the human body (e.g., neurons or hepatocytes, or stem cells that remain quiescent in suboptimal culture conditions). Also the consequences of insertional preference of specific family members close to the transcriptional start site (e.g., gammaretroviruses) or within genes (e.g., lentiviral vectors) need to be further analyzed and it remains to be examined if yet other retroviral vectors (e.g., those based upon the alpharetroviral Rous sarcoma virus [19,20] or human Foamy virus (see review by Lindemann and Rethwilm [210]), which are less biased in their insertion pattern, are beneficial in this regard. Thus, the choice of suitable vector tools should be based on a case-by-case risk benefit assessment, taking into account vector evolution, which—despite promising developments—is still work in progress.

## Figures and Tables

**Table 1.** List of most important pseudotypes, their receptors and envelope modifications required for pseudotyping of MLV to transduce certain target cells / species.

**Pseudotype**	**Abbreviation**	**Receptor**	**Modification**	**Target Species**	**References**
Ecotropic MLV env	Eco	mCAT	not required	mouse and rat	[110]
Amphotropic MLV env	Ampho	PiT2	not required	multiple	[110]
Xenotropic MLV env	Xeno	XPR1	not required	human and others	[98]
Vesicular Stomatitis Virus glycoprotein	VSVg	not determined	not required	multiple	[111]
Simian Endogenous Retrovirus env	RD114	RDR/ASCT2	not required	human and others	[112]
Gibbon Ape Leukemia Virus env	GALV	PiT1	not required	human and ape	[98]
Measles Virus (vaccine strain) H and F proteins	MV	CD150, CD46	not determined	human	[113]
Human Immunodeficiency Virus gp120 env	HIV	CD4 and co-receptor	C-terminal truncation	human	[114]

**Table 2.** Selected gene therapy trials targeting the hematopoietic system or skin disorders.

**Disease**	**Phenotype**	**Affected Gene(s)**	**Conventional Therapy**	**Target Cells**	**Vector**	**Vector Related Side Effects**	**Reference**
Epidermolysis bullosa	Detaching skin and mucosal tissues	Keratin or collagen	Skin graft transplantation	MSC	GV-LTR	n.a.	[89]
Wiskott- Aldrich Syndrome	Immuno deficiency, thombocytopenia and blood cancer	WAS	Drug therapy and / or BM transplantation	HSC	GV-LTR	Leukemia	[162]
Chronic granulomatous disease	Lack of phagocytic lymphocytes	gp91-phox	BM transplantation	HSC	GV-LTR	Leukemia	[161]
SCID-X1	Lack of T and NK cells, and of mature B cells	IL2γc	BM transplantation	HSC	GV-LTR	Leukemia	[86,159,174,180]
SCID-ADA	Immunodeficiency, skeletal and neurologic abnormalities	Adenosine deaminase	Drug therapy and / or BM transplantation	HSC	GV-LTR	n.a.	[160,181]
Beta thalassemia	Anemia	Beta-globin	Blood transfusion	HSC	LV-SIN	Clonal dominance	[182]
Adrenoleuko-dystrophy	Cerebral demyelination	ALD	BM transplantation	HSC	LV-SIN	n.a.	[177]
Melanoma	Skin cancer	various	Dissection of the tumor, chemo- or radiotherapy	T-cells	GV-LTR	n.a.	[183,184]
Graft *versus* host disease	Various	n.a.	Drug therapy	T-cells	GV-LTR	n.a.	[185]
HIV-Aids	Immunodeficiency	n.a.	HAART	HSC, T-cells	LV-LTR	n.a.	[186]
